# Efficacy of a self-guided online resilience intervention for improving mental health among university students: A randomized controlled trial

**DOI:** 10.1038/s41598-026-67088-7

**Published:** 2026-09-02

**Authors:** Sonja Bartelt, Tabea Werner, Lisa von Boros, Sarah K. Schäfer, Friederike Koehler, Benedikt E. Wirth, Klaus Lieb, Oliver Tüscher, Michèle Wessa

**Affiliations:** 1 Institute of Psychology, Johannes Gutenberg University, Mainz, Germany; 2 Department of Neuropsychology and Resilience Research, Central Institute of Mental Health, Mannheim, Germany; 3 Leibniz Institute for Resilience Research (LIR), Mainz, Germany; 4 Clinical Child and Adolescent Psychology and Psychotherapy, RPTU University Kaiserslautern-Landau, Landau, Germany; 5 Department of Music, Art and Culture Studies, University of Jyväskylä, Centre of Excellence in Music, Mind, Body and Brain, Jyväskylä, Finland; 6 German Research Center for Artificial Intelligence (DFKI), Department of Cognitive Assistants, Saarbrücken, Germany; 7 Department of Psychiatry and Psychotherapy, Johannes Gutenberg University Medical Center Mainz, Mainz, Germany; 8 Department of Psychiatry, Psychotherapy and Psychosomatic Medicine, University Medicine of the Martin-Luther University Halle-Wittenberg, Halle, Germany; 9 German Center for Mental Health (DZPG), Site Halle-Jena-Magdeburg, Halle (Saale), Germany; 10 DKFZ Hector Cancer Institute at the University Medical Center Mannheim, Mannheim, Germany; 11 German Cancer Research Center (DKFZ), Division of Cancer Survivorship and Psychological Resilience (C160), Heidelberg, Germany; 12 German Center for Mental Health (DZPG), Site Mannheim-Heidelberg-Ulm, Mannheim, Germany

**Keywords:** Learning Management System, Primary prevention, Mental health, University students, Online interventions, Diseases, Health care, Psychology, Psychology

## Abstract

Epidemiological data indicates that university students are an at-risk population for the development of mental disorders. Online interventions have been proposed as promising tools for reducing barriers to treatment and establishing easily accessible health-care services promoting mental health and resilience. This study investigated the efficacy of a Learning Management System (LMS)-based self-guided online resilience intervention. 216 university students took part in a randomized controlled trial with an intervention and a waitlist control group and three measurement points (pre, post and follow-up). We conducted per-protocol (PP) and intention-to-treat (ITT) analyses with mental distress as primary outcome, and self-reported resilience and resilience factors as secondary outcomes. Further, attitudes towards online interventions, adherence, satisfaction and possible negative effects were explored. Satisfaction with the intervention was high and PP analyses (*n* = 150) revealed significant improvements in mental distress and self-compassion at post-test and acceptance at follow-up. No favourable effects were found for self-reported resilience and resilience factors. Adherence was low and ITT analyses revealed no significant effects. Overall, the study provides preliminary evidence for the LMS-based self-guided online intervention as a potentially valuable tool for university mental health services under optimal adherence conditions. Further research into determinants of adherence is needed to improve intervention reach.

**Supplementary Information:**

The online version contains supplementary material available at https://doi.org/10.1038/s41598-026-67088-7.

## Introduction

University students have been identified as an at-risk population for the development of mental health problems^1,2^ as the prevalence of mental disorders among German university students amounts to 22%^3^. Yet the burden of distress extends beyond formal diagnoses: 87% of German students report feeling stressed sometimes or even often, with the proportion of those who often feel stressed having nearly doubled from 23% to 44% between 2015 and 2023^3^. Another study conducted in Germany indicates that 24% of students reported severe exhaustion, 55% low levels of well-being and 58% low stress resilience^4^. Given this prevalence as well as substantial gaps in health care utilization among university students, it is essential to promote successful adaptation to stress in this population—that is, to foster resilient responses^5^. In this study, resilience is defined as the outcome of a dynamic process of successful adaptation to adversity^6^. Accordingly, resilience is operationalised as the maintenance or recovery of good mental health following adverse life events or periods of challenging life circumstances. Following the definition of resilience as a result of a dynamic process, psychological mechanisms and traits that enable individuals to successfully adapt to adverse events (so-called resilience factors and mechanisms) can be trained in resilience interventions^6–11^. Thereby, the concept of resilience offers great potential for primary prevention and early interventions to improve mental health and prevent mental illness in students^2,6^.

At present, there is no empirically validated consensus framework discerning what constitutes an effective resilience intervention^12^. However, most resilience interventions focus on supporting resilience by fostering one or several health-related resilience factors through approaches adapted from psychotherapy (e.g., cognitive-behavioural therapy, acceptance and commitment therapy or mindfulness-based therapy)^6,9,13–18^. Resilience factors are internal and external resources that buffer negative effects of stressors and are associated with resilient outcomes. Resilience mechanisms are understood to be those methods or strategies that enable individuals to successfully cope with difficult situations^6^ when acutely exposed to stress^19^. It is assumed that resilience factors, such as optimism, self-efficacy, or meaning or purpose in life, influence the activation of respective resilience mechanisms during stressor exposure, thereby contributing to successful adaptation to stress^20^. For example, it is assumed that high self-efficacy expectations in challenging situations lead to relatively positive appraisals of the situation, which prevent overreactions and leave room for a flexible approach and the necessary rebuilding of resources.

Several systematic reviews^9,18,21^ and meta-analyses^16,17,22–25^ have demonstrated small to moderate favourable effects of resilience interventions on resilience and mental health outcomes (e.g., depression, anxiety) in various clinical and non-clinical populations. Among higher education students, a recent meta-analysis^26^ found promising effects of resilience interventions supporting resilience and reducing depressive as well as stress symptoms. Further, a systematic review and network meta-analysis^23^ indicated that psychotherapy, psychoeducation, mindfulness, and skill training significantly support resilience among young adults.

Considering limited help-seeking behaviour among university students, self-guided online interventions represent a promising tool for reducing barriers to treatment and providing low-threshold access to self-help for students who cannot be reached by traditional mental health services^27–34^. Online interventions might be especially appealing to university students who are immersed in the digital world^35^ and are likely to have high digital literacy^22^. Online interventions offer the advantage of high scalability, cost-effectiveness, accessibility regardless of time and location and the possibility to update interventions promptly according to new findings and evidence^36^. Since those interventions can be anonymously accessed, they might also reduce stigma towards mental health and seeking professional help^29,32,35,37–41^.

Although limited in number, previous studies provide preliminary evidence for the efficacy of online resilience interventions among university students, demonstrating significant improvements in resilience and well-being as well as reductions in anxiety and depression symptoms^39,42^. Additional support for online resilience interventions is provided by recent meta-analyses^22,36^ that showed improvements in self-reported resilience as well as mental distress, anxiety and depression, positive mental health, and resilience factors among the general population including university students.

However, further studies are needed to examine the effects of online interventions on resilience and mental health in more detail^22,24,36,37,43^. As systematic reviews on previous resilience intervention studies^9,37,44^ revealed a number of conceptual and methodological weaknesses, Chmitorz and colleagues^44^ propose an outcome-oriented definition and assessment of resilience as change in mental health in relation to stressor exposure, and methodological standards for suitable study designs, such as high psychometric quality of measures, randomization, assessment of satisfaction and adverse events as well as unbiased reporting of study results.

This study aims to contribute to the growing research on online resilience interventions by evaluating the efficacy of a self-guided online intervention (*Resitra*) among university students. The intervention was delivered via the learning management system (LMS) Moodle to engage directly with the students’ everyday lives. Given the findings on resilience interventions and preliminary evidence for online interventions, we expected that *Resitra* will decrease mental distress after controlling for stressor exposure, which is in line with the operationalization of resilience as the maintenance of mental health despite stressor exposure. Further, we assessed a broader set of secondary outcomes including resilience, operationalized as the self-reported ability to bounce back, stress-perception and targeted resilience factors (i.e., optimism, mindfulness, sense of coherence, meaning, acceptance and self-compassion), possible moderators of intervention effects (i.e., intervention adherence and attitudes towards online interventions), satisfaction with the intervention and negative or side effects of the intervention.

## Methods

### Study design and procedure

To test the efficacy of the *Resitra* intervention we conducted a two-armed, randomised controlled trial with three measurement points: baseline (t_0_), post-intervention (t_1_: nine weeks after baseline, i.e., after completion of the last module of the intervention) and follow-up assessment (t_2_: 21 weeks after baseline, i.e., 12 weeks after completion of the intervention). The study was designed and implemented according to the checklist for planning and conducting resilience intervention studies^44^. The trial will be reported in accordance with the CONSORT guidelines for transparent reporting of randomized controlled trials^45^ and the recommendations for internet intervention trials^46^. CONSORT and TIDieR^47^ checklist can be inspected in the Supplement S8.

We recruited all participants as a convenience sample at the Johannes Gutenberg University, Germany in March 2023 by contacting respective study offices via e-mail and using the participant pool MABELLA. Additionally, personal contacts, public notices at the university as well as social media channels were used to inform eligible participants about the study. Participants were only included after receiving detailed information about the study (e.g., aim, procedure, voluntary nature, potential risks, data processing) and giving written informed consent. Further, participants were informed that neither the study’s assessments nor the intervention replaced professional medical or therapeutic diagnosis and care. They were advised to seek professional help in case of critical (mental) health conditions and received information on (local) health-care services. If participants indicated suicidal tendencies in the PHQ-9^48^, they were contacted via email/phone by psychologists of the study team, received information on suitable health services via email (e.g., crisis hotline) and were advised to seek professional help in case of symptom deterioration. The risk of adverse effects while completing the questionnaires and the online intervention was considered low. Regardless, participants were able to withdraw from the study at any time without any disadvantages and giving reason. The participants’ data was pseudo-anonymized using an individual identification number to maintain confidentiality. The study received ethical approval by the Institutional Review board of the Institute for Psychology of Johannes Gutenberg University (2023-JGU-psychEK-005, 10 February 2023) and follows the guidelines of the Declaration of Helsinki^49^. It was registered at the German Clinical Trials Register (Trial Number: DRKS00039378, registration date 02/03/2026).

All data collection took place online via SoSci Survey^50^. After providing informed consent, all eligible participants received questionnaires on sociodemographic variables, stress, resilience, mental health-related constructs and several resilience factors as part of the baseline measurement. In case of non-response, registered participants received up to two reminders via email within two weeks after the invitation for the assessment. The baseline measurement was conducted within two weeks between the 13th and 26th of March 2023. After completing the questionnaire, participants were randomly assigned into either a waitlist control group (CG) or the intervention group (IG) using an allocation ratio of 1:1. Randomization was performed using a computer-generated random number sequence produced by Microsoft Excel’s RAND() function, which generates uniformly distributed pseudorandom numbers. Participants were ranked by their assigned random number and allocated to conditions accordingly. Due to the nature of the intervention, no blinding of participants or researchers was involved. Participants were informed about their group allocation via email. Subsequently, the IG received access to the online training, which was provided via the LMS of the university. The intervention was designed to be completed over eight weeks (27th of March to 21 st of May 2023) and comprised eight modules, each lasting 45–60 min. At the beginning of each week, the participants gained access to a new training module. Each module contained psycho-educational elements and practical exercises focusing on resilience, stress and specific resilience factors such as mindfulness and optimism (see also Supplement S1). At the beginning of each session, participants were asked to complete a short weekly questionnaire through which they could give feedback on the previous training module and rate their current level of self-reported resilience and perceived stress (for results see Table 3; Fig. 2). Mid-week, the participants who did not complete all exercises received a reminder via email to finish this week’s session to support adherence to the intervention. After completing the intervention, outcome measures were reassessed in the IG and CG. The post-assessment was conducted within two weeks after the intervention between the 22nd of May and the 4th of June 2023. The follow-up measurement took place three months after the end of the intervention between 21st of August and the 4th of September 2023. Participants of the CG gained access to the intervention after completing the follow-up measurement. Participants of both groups received monetary compensation for participation (€11 per survey) at each measurement. Psychology students were able to choose course credits as compensation instead (1 h per survey).

### Study sample

**Sample size calculation.** During the design phase of the study, it was planned to conduct covariance analyses (ANCOVA) to analyse the data, and we based our sample size calculations on this choice. We calculated the targeted sample size with G*Power analysis^51^ assuming an α-level of 0.05 and 90% power. The expected effect size was indicated based on previous studies that found moderate effect sizes for the primary outcome mental distress in online intervention studies^22,39^ leading to a minimum sample size of *n* = 74 per group (*N* = 148). As high rates of dropouts of more than 40%^52^ are common for online interventions, we recruited more participants than required (*N* = 261). We deviated from our original plan for the statistical analyses, as strict assumptions must be met for ANCOVA with repeated measurement data, including complete data for all measurement times, sphericity, independence of residuals, and homogeneous regression slopes. These requirements were not met in our data set. We therefore decided to use linear mixed models (see below).

**Inclusion and exclusion criteria.** To be eligible for the study, participants needed to be aged ≥ 18 years and have sufficient knowledge of the German language. Additionally, they had to be enrolled at the local university to receive access to the universities’ online learning platform through which the online intervention was provided and have access to an internet-enabled device with a large screen (tablet, laptop, computer) due to the online setting. Participants currently receiving psychiatric or psychotherapy treatment were excluded for two reasons. First, concurrent treatment constitutes an uncontrolled variable that undermines the ability to attribute outcome changes to the study intervention, thereby threatening internal validity^53^. Second, *Resitra* is grounded in a preventive rather than therapeutic framework, targeting students with subclinical distress and supporting resilience before mental disorders develop. Students currently in treatment are therefore not the primary target group of this intervention and therefore not the adequate target group for testing the efficacy of the resilience training. In a similar manner, students were ineligible if they had an acute mental health crisis (e.g., suicidal tendencies) or were diagnosed with a severe mental illness like schizophrenia or other psychotic disorders, bipolar disorder, and post-traumatic stress disorder.

**Fig. 1:**
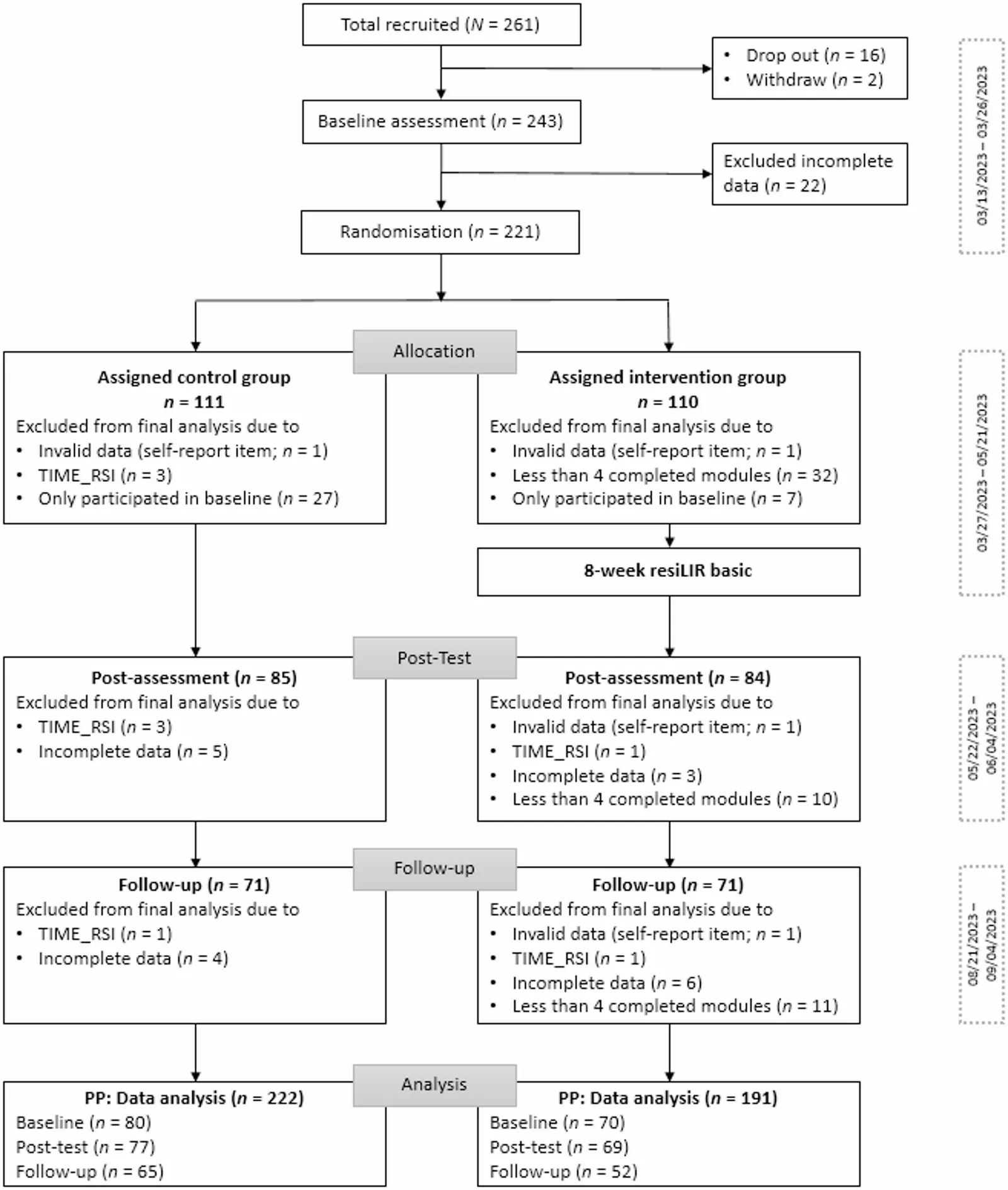
Participant flow through the study, including group allocation, exclusions, and assessment completion rates across all time points. Participants were randomized into IG and CG after baseline completion. Note. For ITT analyses, all available, eligible data was used. With imputing missing values this led to final sample sizes of *n* = 216 per measurement point (IG: 109, CG: 107) which reflects eligible baseline data.

**Sample characteristics.** In total, we recruited 261 students, of whom 221 completed the baseline-assessment. Those participants were randomly assigned to the IG (*n* = 111) and the CG (*n* = 110). Overall, we collected 556 data sets across pre-, post- and follow-up measurement. However, we excluded data sets from the final analyses if the respective survey was not fully completed (*n* = 31), due to not fulfilling the inclusion criteria (*n* = 9), due to self-rated bad data quality (“Did you fill out the questionnaires in a meaningful way? Yes/No; *n* = 4) or excessively fast response times (*n* = 9), as identified by SoSci Survey’s automated quality control indicator (TIME_RSI: relative speed index > 2^54^). This led to 216 baseline measurements, 158 post-tests and 129 three-month follow-ups, which were included in the statistical analyses. Participant flow through the trial is presented in Fig. 1. For an overview of sociodemographic characteristics of the full sample as well as IG and CG see Table 1. To explore possible differences between the IG and CG at baseline, we performed *t*-tests for independent samples and χ2-tests. There were no significant differences between the groups regarding sociodemographic and baseline variables (e.g., psychological distress, attitudes towards online interventions) at baseline except for study course (χ2(12) = 23.21, *p* =.026).

**Table 1:** Demographic and study-related information of participants at baseline.

	*All (n = 216)*		*IG (n = 109)*		*CG (n = 107)*			t-test/χ^2^-test
	M [95%-CI]	Range	M [95%-CI]	Range	M [95%-CI]	Range	*p*	
Age	23.75 [23.27; 24.22]	19–49	24.03 [23.30; 24.75]	19–49	23.46 [22.83; 24.08]	19–37	0.242	
	*N*	%	*N*	%	*N*	%	*p*	
Gender							0.738	
Female	176	81.48	91	83.49	85	79.44		
Male	38	17.59	17	15.60	21	19.63		
non-binary	2	0.93	1	0.92	1	0.93		
Education							0.245	
University entrance qualification (German: Abitur)	141	65.28	66	60.55	75	70.09		
Bachelor	65	30.09	39	35.78	26	24.30		
Master	6	2.78	3	2.75	3	2.80		
First state exam	4	1.85	1	0.92	3	2.80		
Faculty at Johannes Gutenberg-University							0.029	
Catholic and Protestant Theology	2	0.93	0	0	2	1.87		
Social Sciences, Media, and Sports	72	33.33	35	32.11	37	34.58		
Law, Management and Economics	48	22.22	27	24.77	21	19.63		
Medicine	21	9.72	9	8.26	12	11.21		
Philosophy and Philology	25	11.57	17	15.60	8	7.48		
Translation Studies, Linguistics, and Cultural Studies	2	0.93	1	0.92	1	0.93		
History and Cultural Studies	7	3.24	5	4.59	2	1.87		
Physics, Mathematics, and Computer Science	10	4.63	1	0.92	9	8.41		
Chemistry, Pharmaceutical Sciences, Geography and Geosciences	12	5.56	4	3.67	8	7.48		
Biology	10	4.63	6	5.50	4	3.74		
Mainz School of Music	3	1.39	3	2.75	0	0		
Mainz Academy of Fine Arts	1	0.46	1	0.92	0	0		
Other	3	1.39	0	0	3	2.80		

Note. All data from baseline measurement. IG = Intervention group; CG = Waitlist control group; *M* = mean; *SD* = standard deviation; *p* = *p* value (based on *t*-tests and χ^2^-tests). PP analyses included fewer participants due to dropout and non-adherence to the intervention. Descriptive data of the PP sample can be inspected in Supplement S3.

### Intervention

The *Resitra* intervention was developed by the authors at the Leibniz Institute for Resilience Research as part of the APPWAG project (“Ausbau online-basierter Programme zur Resilienzförderung zu einer Plattform für die Weiterentwicklung von zielgruppenspezifischen Angeboten der Gesundheitsförderung”). This project aimed at developing and evaluating a low-threshold evidence-based online training program to foster individual resilience and to improve mental health in the general public^55^ by targeting selected resilience factors (i.e., optimism, mindfulness, sense of coherence, meaning, acceptance and self-compassion) in the intervention. Accordingly, the self-guided online intervention incorporates elements of Cognitive Behavioural Therapy, Mindfulness-Based Stress Reduction, Acceptance and Commitment Therapy, Compassion Focused Therapy and Positive Psychology. For an overview of the content of each session, please see Supplement S1. The program consists of a combination of animated explanatory videos, audios and texts as well as interactive exercises. Integrated transfer tasks were included to strengthen the application of newly acquired skills, alongside reflective questions prompting participants to consider how and when to apply skills in their daily lives.

### Outcome variables and measures

In the following, all questionnaires relevant for the present study are presented. All questionnaires were administered in German, and were included in the pre-, post- and follow-up assessment except for *attitudes towards psychological online interventions* (only baseline), *satisfaction with intervention* and *negative effects of psychological online interventions* (only IG at post-test). We calculated Cronbach’s and McDonald’s *ω* as measures of reliability of the respective questionnaires. Reliability checks demonstrated sufficient internal consistency for all outcome measures (Cronbach’s α >= 0.63; McDonald’s *ω* >= 0.63; see Supplement S2 for details).

### Primary outcome

**Mental distress**. We measured mental distress using the German version of the General Health Questionnaire-12 (GHQ-12)^56^. The GHQ-12 asks participants about how they felt in the past two weeks relative to how they normally feel and measures the intensity of internalizing symptoms (e.g., ability to concentrate, quality of sleep, decision making) based on 12 items rated on a 4-point Likert scale with an item scoring from 0 (*not at all/better than usual)* to 3 (*much more than usual*/*much worse than usual)*. A sample item includes: “Have you been able to adequately cope with your problems?”. A total mental distress score was computed by calculating a sum score of the 12 mental distress items. Therefore, higher values indicate greater mental health problems.

### Secondary outcomes

**Resilience.** We used the 6-item Brief Resilience Scale (BRS)^57^ to assess self-reported resilience as “bouncing back from adversity”. Participants rated their agreement with statements on recovery from difficult situations on a 5-point Likert scale ranging from 1 (*strongly disagree*) to 5 (*strongly agree*). After reverse-coding three items, higher total scores indicate greater resilience. Moreover, we asked participants at the beginning of each module to indicate how resilient they are right now on a 10-point Likert scale ranging from 1 (*not very resilient*) to 10 (*very resilient*).

**Perceived stress**. We measured perceived stress with the Perceived Stress Scale 2&2 (PSS 2&2)^58^, which consists of two 2-item subscales measuring self-efficacy and helplessness. Participants were asked to rate how often they had experienced stress-related thoughts and feelings during the past month on a 5-point Likert scale from 1 (*never*) to 5 (*very often*). Mean scores were computed for each subscale; higher helplessness scores indicate greater perceived stress, whereas higher self-efficacy scores indicate lower perceived stress. Additionally, we asked participants at the beginning of each module to indicate how stressed they are right now on a 10-point Likert scale ranging from 1 (*not stressed at all*) to 10 (*very stressed*).

### Targeted resilience factors

**Optimism.** Optimism was measured as a trait by the Optimism-Pessimism Scale-2 (SOP-2)^59^. This self-assessment scale includes two items assessing how optimistic/pessimistic participants are in general and rated on a 7-point Likert scale from 1 (*not at all optimistic/pessimistic*) to 7 (*very optimistic/pessimistic*). Before computing the total score, Item 2 was recoded so that high values represent dispositional optimism. A total mean score of both items was calculated.

**Mindfulness**. Mindfulness was assessed using the Mindful Attention and Awareness Scale (MAAS-Short)^60^. It measures mindfulness by 10 items that relate to everyday experiences (e.g., “I do jobs and tasks with awareness”) and rated on a 6-point Likert scale ranging from 1 (*almost always*) to 6 (*almost never*). To simplify interpretation, items were recoded prior to further analyses so that high values correspond to a high level of mindfulness. A total mean score was calculated.

**Sense of coherence**. The Sense of Coherence Scale-29 (SOC-29)^61^ was used to assess the resilience factor sense of coherence. Participants were asked to indicate their agreement on 29 items with individual 7-point Likert scales that vary between items. A sample item includes: “How often do you have the feeling that there’s little meaning in the things you do in your daily life?” (1–7; 1 = *very often*, 7 = *very seldom or never*). For the analysis, some item ratings were recoded so that high ratings always correspond to high levels of sense of coherence. Then, a total mean score was calculated.

**Meaning**. Along with the sense of coherence, the participants’ perception of meaning in life was assessed. This perception was measured using the subscale Meaning of the Comprehensive Inventory of Thriving (CIT)^62^ which comprises three items (e.g., “My life has a clear purpose”) that are rated on a 5-point Likert scale ranging from 1 (*strongly disagree*) to 5 (*strongly agree*) with higher values indicating higher perceived meaning in life. An overall mean score of all three items was calculated.

**Acceptance**. The corresponding 3-item subscale of the Cognitive Emotion Regulation Questionnaire (CERQ)^63^ was used to assess acceptance. The participants indicated on a 5-point Likert scale ranging from 1 (*(almost) never*) to 5 (*(almost) always*) what they generally think when they experience an unpleasant event and how often they use acceptance as an emotion regulation strategy with higher scores indicating more frequent acceptance. A sample item includes: “I think I have to accept that this has happened”. An overall mean score of all three items was determined.

**Self-compassion**. Self-compassion was assessed as a trait by the German version of the Self Compassion Scale (SCS-D)^64^. The questionnaire comprises six subscales (self-kindness, self-judgement, common humanity, isolation, mindfulness, over-identification) and a total of 26 items (e.g., “I try to be loving towards myself when I’m feeling emotional pain”) that are rated on a 5-point Likert scale ranging from 1 (*almost never*) to 5 (*almost always*). Negative subscale items (self-judgement, isolation, over-identification) were recoded so that high scores indicate high self-compassion. Afterwards a mean of the six subscales was calculated as a total self-compassion score.

### Covariates and moderators

**Stressor exposure**. Stressor exposure was measured by the Mainz Inventory for Micro Stressors (MIMIS)^65^. The MIMIS allows the retrospective assessment of micro stressors (daily hassles) and their perceived severity over a one-week period. The scale includes a list of 58 stressors like financial stress, interpersonal conflicts (e.g., family, friends, colleagues) and stressors (e.g., lack of social support, discrimination) as well as academic pressure (e.g., high workload, exams) which are rated according to frequency of their occurrence (0–7; 0 = *did not occur*, 7 = *occurred every day*) and perceived stressor severity (0–4; 0 = *not at all severe*, 4 = *extremely severe*) during the last seven days. The MIMIS was analysed by the means of a stressor-count-method in which reported daily hassles are counted by adding up the numbers of days that each item occurred. A total sum score over all reported stressors was calculated (max = 406). The stressor count method was chosen because it reflects the mere occurrence of stressors, while not being confounded by individual differences in the way stressors are perceived. Therefore, we did not include stressor severity ratings in the analyses.

**Attitudes towards psychological online interventions**. We used the German Attitudes towards Psychological Online Interventions Questionnaire (APOI)^66^ at baseline to assess participants’ attitudes towards psychological online interventions. The APOI contains the subscales *Confidence in Effectiveness* (e.g., “can help me to recognize the issues that I have to challenge”), *Anonymity Benefits* (e.g., “I can reveal my feelings more easily than with a therapist”), *Scepticism and Perception of Risks* (e.g., “I do not expect long-term effectiveness”) and *Technologization Threat* (e.g., “In crisis situations, a therapist can help me better”). On a 5-point Likert scale ranging from 1 (*strongly disagree*) to 5 (*strongly agree*) the participants rated 16 items according to their agreement. The total score includes the mean of all positive subscale items and recoded negative items. Hence, a higher total score represents a more positive attitude towards psychological online interventions.

**Satisfaction with intervention**. We assessed the satisfaction twofold: First, we used the Client Satisfaction Questionnaire for Internet-based interventions (CSQ-I)^67^. The participants indicate their agreement with eight items (e.g., “The intervention met my needs”) on a 4-point Likert scale ranging from 1 (*does not apply*) to 4 (*does totally apply*) with higher scores indicating higher satisfaction with the intervention. Second, we asked participants at the beginning of each module to indicate their satisfaction with the last module on a 10-point Likert scale ranging from 1 (*not at all satisfied*) to 10 (*very much satisfied*).

**Negative effects of online interventions.** In the IG we assessed adverse effects of the online intervention with the Inventory of Negative Effects of Psychotherapy for Online-Interventions (INEP-ON)^68^. Participants rate in total 23 items (e.g., “Since completing the online intervention, I feel… worse/better”). Of these, items 1 to 11 are rated on a 7-point scale ranging from − 3 (negative outcome) to + 3 (positive outcome); items 12 to 21 are rated on a 4-point scale ranging from 0 (*fully disagree*) to 3 (*fully agree*). Each item is rated whether this change is attributed to the intervention or life circumstances. Items 22 and 23 are also rated on a 4-point scale ranging from 0 (*fully disagree*) to 3 (*fully agree*). We report item mean and standard deviation.

**User statistics.** Regarding the intervention, the number of processed modules and the feedback questionnaires within the intervention platform (e.g., if exercises were adopted in daily life) were analysed. To determine intervention adherence, a sum score of completed modules was calculated (max = 8). Within feedback questionnaires, the frequency of exercises transferred into daily life was either answered yes/no at the beginning of each new module or rated on a 5-point Likert scale ranging from 1 (almost never) to 5 (almost always) after the end of the whole intervention.

### Data preparation and analyses

We conducted all data preparation and analyses with R^69^ and RStudio^70^. Data of the present study and the respective analysis script necessary to replicate the analyses are available online *(*https://osf.io/mtkw7/overview?view_only=d13a5e6bad3d475985ce0d40315ed85f*).* For all inference statistics calculations, we set an error probability of α = 0.05 a priori.

**Intervention adherence**,** satisfaction**,** and negative effects.** Besides investigating intervention effects, we descriptively and qualitatively analyzed intervention adherence, transfer of exercises into daily life, satisfaction with the intervention, and potential harmful effects.

**Intervention efficacy.** Our analyses were conducted in accordance with established recommendations for the analysis of randomized controlled trials^71,72^. To estimate the intervention effect on targeted outcomes over time (t_0_-t_2_), we conducted linear mixed models (LMM) with the lme4 package^73^. LMM address the clustered structure of data as repeated measures (time) are nested within participants (subjects), and participants are nested within groups. Disregarding the clustered structure would result in biased estimates of standard errors and potentially increase the risk of type I error. LMM have been suggested as an appropriate method in the analysis of clustered data structure and modelling temporally correlated data^74^. To test model assumptions, the residuals from each model were examined for normality using QQ-Plots and Shapiro-Wilk tests. Shapiro-Wilk tests revealed some violations of distributional assumptions. However, based on previous simulation studies^75–77^, linear mixed models are largely robust to violations of modelling assumptions frequently observed in real-world data, given a total sample size of 45 or larger. Further, the assumption of homogeneity of variance was supported based on non-significant findings of Levene-tests. In assessing intervention effects, we conducted both, per-protocol (PP) and intention-to-treat (ITT) analyses. PP analyses were defined as primary to examine intervention effects among participants with sufficient engagement, while ITT analyses were conducted as sensitivity analyses for conservative intervention effect estimates. In the PP analysis, we only included participants who took part in post- and/or follow-up assessment of outcome measures. Further, only those participants of the IG who completed at least four out of the eight intervention modules (50%) were included in the analyses. To handle missing questionnaire data for ITT analyses, we used multiple imputation as implemented in the mice^78^ package. When applicable, we used multilevel predictive mean matching (2 L.pmm) to account for hierarchical data structures. This method allows for the imputation of hierarchically structured longitudinal data by accounting for between-person and within-person variability, resulting in multiple imputed datasets that preserve the nested structure of repeated measures. Otherwise, we used standard predictive mean matching (pmm). We generated 20 imputed datasets and pooled estimates according to Rubin’s rule^79^.

The primary outcome was mental distress (GHQ-12) controlled for stressor exposure (MIMIS). In addition to that, we added a proximal measure of resilience, the Brief Resilience Scale, which conceptualizes resilience as the ability to recover from stress, and perceived stress (PSS 2&2) as secondary outcomes, since these measures are widely used in intervention research, rendering our study results comparable to past and future studies. Finally, we investigated resilience factors targeted in the intervention. As recommended by Twisk^80^, we set up two models for each outcome and analysed the data using repeated measures analysis with baseline adjustment. First, we fitted a linear mixed model including all measurements (baseline, post-intervention and follow-up) to estimate the overall intervention effect. Models included time (coded 0 for baseline and 1 for all following assessments) as a fixed effect, the interaction between time and group (coded 0 for CG and 1 for IG) and a random intercept for participants to account for repeated measurements. To adjust for baseline differences, the main effect of group was not included in both models constraining models to equal baseline means across groups. The time x group regression coefficient here indicates the overall intervention effect, namely the difference between the IG and CG on average over time. Second, time-specific intervention effects were examined by introducing dummy variables for post-intervention (week 9) and follow-up (week 21), yielding baseline-adjusted estimates of group differences at each time point. The group x time dummy coefficients indicate intervention effects at the respective time points. In all models, we included age and gender as covariates. Additionally, we included stressor exposure as covariate in the model examining the intervention effect on mental distress.

**Moderator analyses.** We performed explorative analyses to examine the influence of two possible moderators on intervention effects in primary and secondary outcomes within the IG: intervention adherence operationalized as completed modules and attitudes towards online interventions. These analyses tested whether participants who received the intervention improved differentially depending on their attitudes and amount of completed modules, but they do not represent moderation of the between-group intervention effect. For these variables, we set up LMMs with random intercepts for participants and time x moderator interactions. Before running the models, we grand-mean centered intervention adherence and attitudes. All moderators were analyzed in separate models.

**Dropout and adherence analyses**. Selective attrition was examined by comparing completers and dropouts (defined by participants without post-intervention data) on baseline characteristics using independent *t*-tests. Furthermore, we investigated whether baseline data significantly predicted intervention adherence (i.e., the amount of completed modules).

**Robustness of results**. As a sensitivity analysis addressing multiple testing, we adjusted *p*-values for secondary outcomes with the Hommel procedure^81^ which controls the family-wise type I error rate while retaining greater statistical power than Bonferroni-based adjustments^82^. We did not adjust *p*-values for time-specific contrasts in secondary outcomes, as we considered them to be a decomposition of the overall effects. The primary outcome was tested at *α* = 0.05 without multiplicity adjustment.

## Results

### Descriptive statistics

Means and standard deviations of all outcomes at baseline, post- and follow-up assessments for both study groups are depicted in Table 2, correlations between primary and secondary outcomes, targeted resilience factors and covariates at baseline are depicted in Supplement 4 in Table S4. *t*-tests for independent samples revealed no significant differences between IG and CG at baseline (*p* >.269).

**Table 2:** Descriptive statistics of primary and secondary outcomes as well as covariates for pre-, post and follow-up data.

	IG						CG							
	t_0_ (*n* = 109)		t_1_ (*n* = 79)		t_2_ (*n* = 63)		t_0_ (*n* = 107)		t_1_ (*n* = 79)		t_2_ (*n* = 66)			
Outcome	*M*	*SD*	*M*	*SD*	*M*	*SD*	*M*	*SD*	*M*	*SD*	*M*	*SD*	*p*	
*Primary outcome*														
Mental distress (GHQ)	14.93	6.88	12.25	5.34	11.41	5.95	14.92	6.66	13.85	6.04	12.15	5.84	0.991	
*Secondary outcomes*														
Resilience (BRS)	3.16	0.81	3.25	0.74	3.31	0.71	3.12	0.73	3.26	0.73	3.38	0.68	0.755	
Perceived stress – self-efficacy	3.54	0.78	3.63	0.71	3.71	0.68	3.50	0.72	3.54	0.73	3.65	0.67	0.753	
Perceived stress – helplessness	3.17	0.82	3.04	0.75	2.89	0.82	3.29	0.79	3.08	0.79	3.02	0.80	0.274	
*Targeted resilience factors*														
Optimism	4.48	1.34	4.58	1.37	4.58	1.23	4.57	1.35	4.51	1.29	4.71	1.15	0.612	
Mindfulness	3.82	0.81	3.87	0.80	3.88	0.80	3.77	0.85	3.77	0.83	3.87	0.81	0.670	
Sense of coherence	4.44	0.78	4.52	0.76	4.54	0.74	4.41	0.75	4.47	0.71	4.61	0.69	0.768	
Meaning	3.53	0.82	3.64	0.81	3.62	0.86	3.48	0.81	3.59	0.85	3.69	0.68	0.677	
Acceptance	4.80	0.93	4.87	0.91	4.85	0.82	4.76	0.85	4.71	0.91	4.56	1.00	0.753	
Self-compassion	3.01	0.70	3.19	0.65	3.20	0.69	2.96	0.68	3.00	0.70	3.08	0.73	0.595	
*Covariates*														
Stressor exposure	70.61	32.12	64.82	38.15	59.79	37.04	74.26	30.38	69.92	39.28	62.11	29.74	0.392	
Attitudes towards online interventions	3.21	0.45	-	-	-	-	3.28	0.43	-	-	-	-	0.269	

Note. IG = intervention group; CG = waitlist control group; two persons from the IG were excluded due to bad data quality (see Method section and Fig. 1 for more information); *p*-values refer to differences at baseline.

**Intervention adherence and dropout**. 13 out of 110 participants (11.82%) completed no module at all whereas 41 (37.27%) finished the whole intervention. On average, participants completed roughly 5 out of 8 modules (*M* = 5.02, *SD* = 2.98) which equals 62.5% of the intervention (Table 3). Women finished significantly more modules than men (*M*_women_ = 5.37, *M*_men_ = 3.59, *t*(22.66) = 2.34, *p* =.029, Cohen’s d = 0.61). Moreover, higher satisfaction with the intervention assessed with the CSQ-I was associated with more completed modules (ß = 0.97, *F*(1, 77) = 5.27, *p* =.024, *R*^*2*^ = 0.06) whereas neither age nor attitudes towards online interventions, mental distress or stressor exposure at baseline significantly predicted the amount of completed modules (*p* >.05). Examining selective attrition in post-intervention data, we compared dropouts (*n* = 30) with participants completing the post-test (completers, *n* = 79)^1^. *t*-tests for independent samples for continuous variables and chi-square tests for categorical variables revealed no significant differences at baseline in mental distress, stressors, age or gender (*p* >.05) but in attitudes towards online interventions with completers having more positive attitudes (*M*_completers_ = 3.29, *M*_dropout_ = 3.01, *t*(53.76) = 3.04, *p* =.004, Cohen’s *d* = 0.64).

**Satisfaction with intervention.** Overall satisfaction with the intervention was high, with a mean satisfaction of *M* = 3.24 (*SD* = 0.59, Range: 0–4) in the CSQ-I and of 7.71 answering a single item. Detailed results from the CSQ-I can be inspected in Supplement S5. Satisfaction with each module is depicted in Table 3. Furthermore, satisfaction with the intervention was also indicated within qualitative feedback which participants of the IG could give via open-ended questions on the intervention platform. Specifically, participants liked the clear structure of modules, the variety of audios, texts and videos as well as practical exercises. Possible suggestions for improvement and further development of the program were *time* (not enough time to complete modules within the recommended week due to other duties), *support* (additional face-to-face contacts would be helpful), *platform* (not suitable as intervention platform, complicated to use, too similar to university courses) and *transfer* (limited transfer of exercises into daily life).

**Transfer to daily life.** Weekly feedback questionnaires indicated that transfer of one or more exercises to daily life ranged between 54% and 77%. However, despite these promising figures, participants indicated in the overall feedback at the end of the intervention that the transfer of exercises into everyday life was limited. From 65 participants answering that question, only 15.38% (*n* = 10) indicated that they often adopted exercises, 63.08% (*n* = 41) sometimes, 13.85% (*n* = 9) rarely and 7.69% (*n* = 5) never.

**Negative effects of online interventions.** Across the 11 items measuring positive and negative intervention effects, 43 participants (54.43%) reported at least one negative effect, whereas 75 (94.94%) reported at least one positive effect. The most common negative effect was increased feelings of loneliness (22.67%) and decreased hobbies and social contacts (16.00%). However, only two or three students attributed the negative effects to the intervention whereas all others attributed them to life circumstances or neither of both. Positive effects attributed to the intervention included learning helpful ways of thinking (86.11%), a supportive guide (78.18%) and feeling better (47.17%). Across the 12 items that measured only negative intervention effects, 53 participants (67.08%) reported at least one mild (*n* = 42), moderate (*n* = 8), or severe (*n* = 5) effect. Importantly, data protection concerns were most frequently cited, with 25 reporting mild to severe concerns. Further, the two remaining participants reporting severe negative effects indicated to find it more difficult to make important decisions on their own or having prolonged periods of feeling bad. Detailed results can be inspected in Supplement S7.

**Fig. 2:**
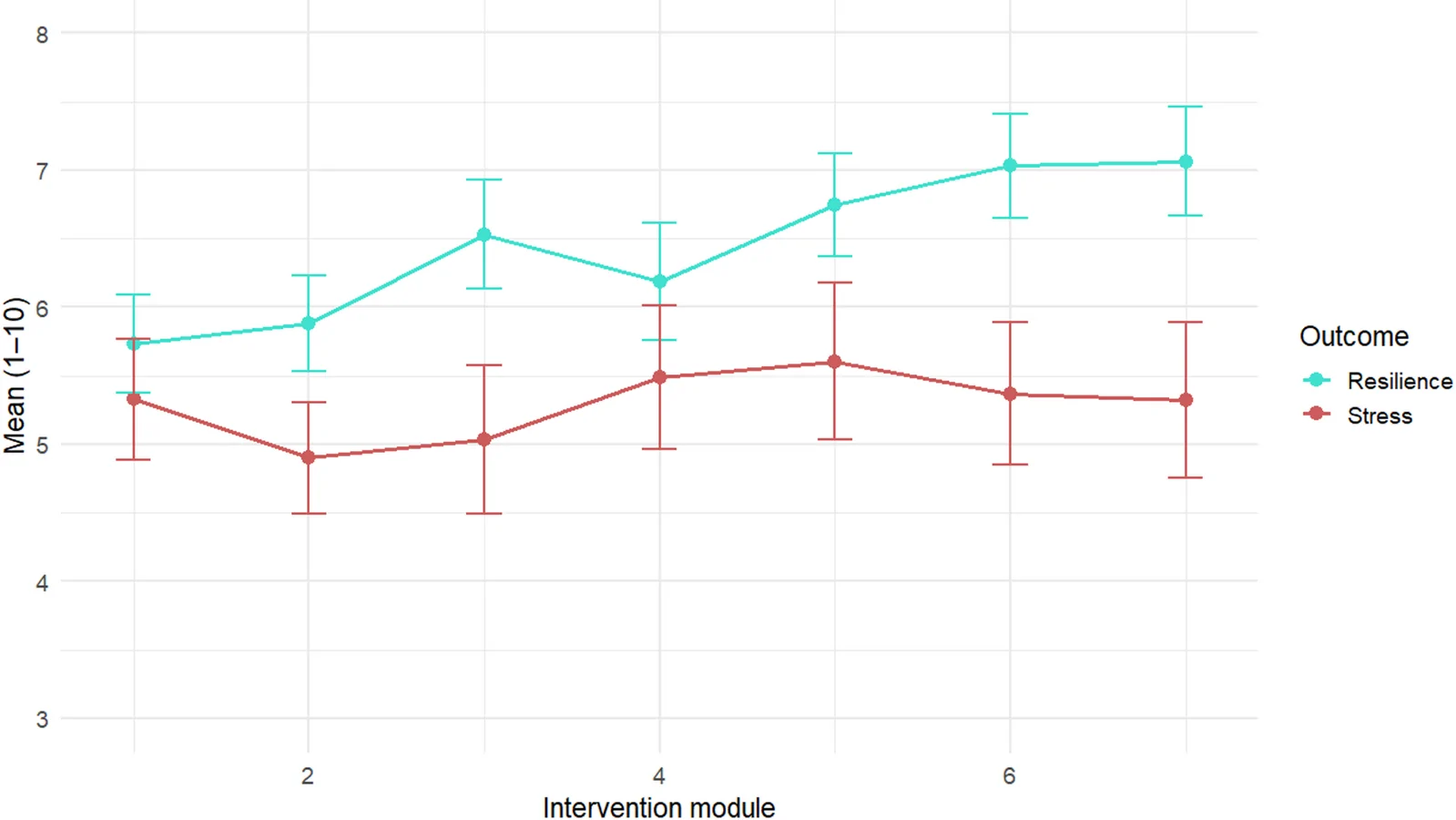
Mean trajectory of self-rated resilience and perceived stress at the beginning of each module with 95% confidence intervals.

**Self-rated resilience and stress.** At the beginning of each module (except introduction) participants indicated their self-rated resilience and perceived stress level. Throughout the intervention, perceived stress remained largely stable, whereas self-rated resilience increased over time (see Fig. 2; Table 3).

**Table 3:** Intervention adherence, satisfaction with modules, transfer to daily life, self-rated resilience and perceived stress (session ratings).

Module	Adherence		Overall satisfaction				Daily use				Self-rated resilience			Self-rated stress		
	*n*	%	*n*	M	SD	Range	Yes	%	No	%	M	SD	Range	M	SD	Range
Introduction	97	88.18	-	-	-	-	-	-	-	-	-	-	-	-	-	-
Resilience and Stress	87	79.09	101	7.65	1.55	[2; 10]	51	56.04	40	43.96	5.73	1.84	[2; 9]	5.33	2.27	[1; 10]
Optimism	81	73.64	83	7.89	1.64	[2; 10]	60	76.92	18	23.08	5.88	1.79	[2; 9]	4.90	2.06	[1; 10]
Mindfulness	77	70.00	81	7.04	2.10	[1; 10]	54	72.97	20	27.03	6.53	1.82	[1; 10]	5.04	2.47	[1; 10]
Values and sense of coherence	62	56.36	71	6.85	2.42	[1; 10]	33	63.46	19	36.54	6.19	1.92	[2; 10]	5.49	2.37	[1; 10]
Acceptance	56	50.91	65	7.74	1.72	[1; 10]	31	54.39	26	45.61	6.75	1.58	[2; 10]	5.61	2.41	[1; 10]
Self-Compassion	48	43.64	63	7.35	2.23	[1; 10]	25	59.52	17	40.48	7.03	1.54	[3; 10]	5.37	2.09	[1; 10]
Conclusion/Overall	41	37.27	65	7.71	2.04	[1; 10]	-	-	-	-	7.06	1.58	[3; 10]	5.32	2.24	[1; 9]

*Note. N* = 110 were assigned to the IG; 13 dropped out immediately, completing no module. Numbers of satisfaction ratings and daily use are higher as it was not necessary to complete the module to give feedback to the previous module. Daily use questions were voluntary. Participants indicated whether they adopted one or more exercises from the last lesson and implemented them in everyday life. *M* = mean, *SD* = standard deviation.

### Intervention effects

**Fig. 3:**
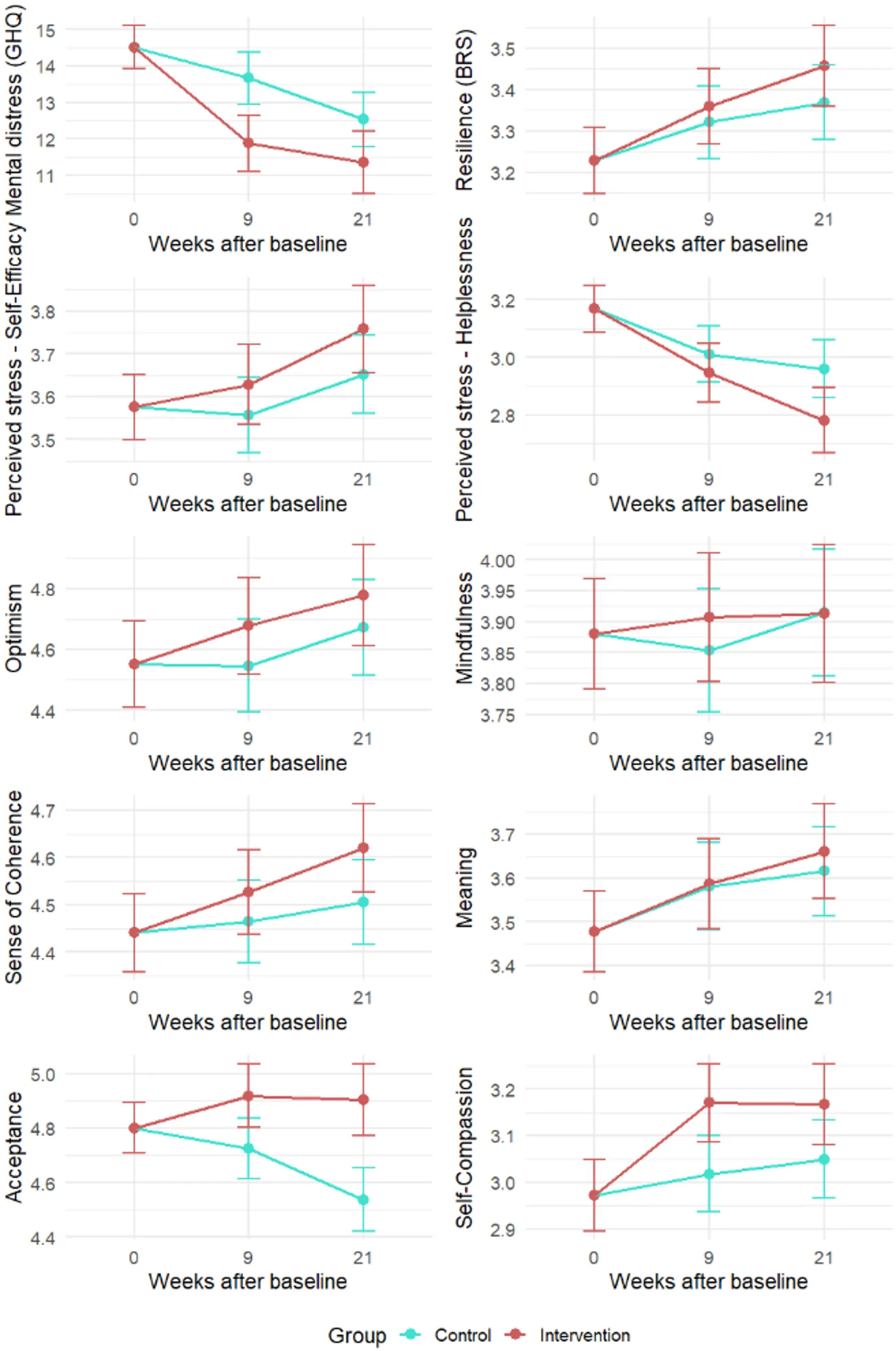
Plots of primary and secondary outcomes of PP analyses. *Note.* Values represent model-based estimated marginal means (EMMs) from a linear mixed-effects model adjusted for baseline. Error bars show 95% confidence intervals.

**PP analysis.** Results of PP analyses are depicted in Table 4 and graphically illustrated in Fig. 3. After controlling for stressor exposure, age and gender, results revealed a significant group x time overall interaction favouring the IG for reducing mental distress, with the effect being significant at post-intervention but not significantly maintained at follow-up. No significant effect could be detected regarding self-reported resilience (BRS). Furthermore, there was a significant group x time overall interaction favouring the IG for increasing acceptance and self-compassion, with the effect being significant at post-intervention but not significantly maintained at follow-up for self-compassion and the other way round for acceptance. However, sensitivity analyses applying Hommel adjustment across secondary outcomes indicated that effects for acceptance and self-compassion did not meet the adjusted significance threshold. Furthermore, there were no significant effects for any other secondary outcome.

**Table 4:** Results of PP analyses of primary and secondary outcomes: Overall group x time effects and specific group x time effects for post-intervention and follow-up.

Outcome		b	SE	95% CI	df	t	*p*_raw_	*p*_Hommel_
*Primary outcome*								
Mental distress (GHQ)	Overall	−1.49	0.73	[−2.91; −0.06]	402.90	−2.05	**0.041**	-
	9	−1.79	0.88	[−3.51; −0.07]	390.99	−2.04	**0.042**	-
	21	−1.18	0.98	[−3.09; 0.74]	382.22	−1.20	0.230	-
*Secondary outcomes*								
Resilience (BRS)	Overall	0.06	0.07	[−0.08; 0.19]	348.41	0.81	0.419	0.787
	9	0.04	0.08	[−0.12; 0.20]	324.47	0.47	0.639	-
	21	0.09	0.09	[−0.09; 0.26]	316.75	1.00	0.319	-
Perceived stress – self-efficacy	Overall	0.08	0.08	[−0.08; 0.25]	397.05	0.99	0.324	0.787
	9	0.07	0.10	[−0.12; 0.26]	365.18	0.74	0.463	-
	21	0.11	0.11	[−0.11; 0.32]	354.72	0.98	0.327	-
Perceived stress - helplessness	Overall	−0.11	0.09	[−0.30; 0.08]	406.37	−1.15	0.249	0.787
	9	−0.06	0.11	[−0.29; 0.16]	379.19	−0.58	0.564	-
	21	−0.18	0.13	[−0.42; 0.07]	369.12	−1.43	0.155	-
*Targeted resilience factors*								
Optimism	Overall	0.12	0.11	[−0.10; 0.33]	330.89	1.04	0.299	0.787
	9	0.13	0.13	[−0.12; 0.38]	311.57	1.03	0.303	-
	21	0.10	0.14	[−0.17; 0.38]	305.30	0.74	0.457	-
Mindfulness	Overall	0.03	0.08	[−0.14; 0.19]	364.34	0.34	0.732	0.787
	9	0.05	0.10	[−0.14; 0.24]	337.38	0.55	0.580	-
	21	−0.00	0.11	[−0.21; 0.21]	328.55	−0.02	0.986	-
Sense of coherence	Overall	0.08	0.05	[−0.02; 0.19]	306.99	1.52	0.130	0.651
	9	0.06	0.06	[−0.06; 0.18]	293.68	1.01	0.313	-
	21	0.11	0.07	[−0.02; 0.25]	289.49	1.71	0.088	-
Meaning	Overall	0.02	0.07	[−0.12; 0.16]	331.61	0.27	0.787	0.787
	9	0.00	0.08	[−0.16; 0.17]	311.97	0.05	0.958	-
	21	0.05	0.09	[−0.14; 0.23]	305.53	0.49	0.622	-
Acceptance	Overall	0.27	0.11	[0.05; 0.49]	407.60	2.44	**0.015**	0.122
	9	0.19	0.13	[−0.07; 0.46]	387.64	1.45	0.148	-
	21	0.37	0.15	[0.08; 0.66]	378.23	2.47	**0.014**	-
Self-compassion	Overall	0.14	0.05	[0.03; 0.24]	315.59	2.56	**0.011**	0.089
	9	0.15	0.06	[0.02; 0.27]	300.64	2.48	**0.014**	-
	21	0.12	0.07	[−0.03; 0.25]	295.65	1.74	0.084	-

Note. Week 9 = post-intervention; week 21 = follow-up (3 months after completion of the intervention). *p*_raw_ = unadjusted *p*-values, *p*_hommel_ = Hommel-adjusted *p*-values (as sensitivity analysis for secondary outcomes). Unadjusted *p*-values < 0.05 are shown in bold.

### Moderator analyses

Exploratory moderator analyses revealed no significant moderating effect of attitudes towards online intervention on any primary or secondary outcome (*p* >.05) neither in the PP nor in the ITT approach. Investigating the moderating effect of completed modules, we conducted analyses only in the ITT-sample with no significant moderating effect except for optimism (Overall: *b* = 0.06 95% CI [0.01; 0.11], *t*(159.73) = 2.23, *p* =.027; Post-test: *b* = 0.06 [0.01; 0.11], *t*(178.72) = 2.10, *p* =.037, Follow-Up: *b* = 0.05 [0.01; 0.11], *t*(119.77) = 1.71, *p* =.089). Participants completing more modules reported greater increases in optimism over time.

### Sensitivity analysis

Results from the ITT analyses revealed no significant intervention effects after adjusting *p*-values. Detailed results can be inspected in Supplement S6.

## Discussion

This study investigated the efficacy of an LMS-based, self-guided online resilience intervention among university students. As expected, when compared to a CG and after controlling for stressor exposure, PP analyses revealed significantly decreased mental distress in the IG. This aligns with previous research that found positive effects of online interventions promoting mental health in university students^28,43,83^. Given the high prevalence of stress-related mental disorders among university students, this finding indicates promising effects for online resilience interventions as a prevention strategy to improve mental health in this population. In contrast to initial expectations, PP analysis revealed no significant effects on resilience measured with the Brief Resilience Scale. Regarding targeted resilience factors, we only observed significant improvements in self-compassion at post-test and acceptance at follow-up. However, after adjusting *p*-values to account for multiple testing, no significant results remained and we observed no significant intervention effects on other targeted resilience factors. Further, no significant intervention effects on primary and secondary outcomes as well as targeted resilience factors were found in ITT analyses. Together with the low adherence to the resilience training (mean attrition rate from one training module to the next was 11% with a total attrition rate of around 63) these diverging results from PP and ITT analyses suggest potential efficacy of the LMS-based resilience intervention only under optimal adherence conditions.

Besides pre-post intervention effects, we also investigated session-by-session effects on self-rated stress and resilience. While self-rated stress remained stable throughout the session-by-session assessment, self-rated resilience increased over the course of the intervention. This indicates that, while there was no significant increase in resilience measured with the BRS when compared with the CG, participants reported improved resilience in the feedback provided during the intervention. One possible explanation for this, is that the BRS assesses individuals’ general and relatively stable self-perceived capacity to recover from stress, representing a more trait-like measure. Therefore, it might be less sensitive to short-term changes following resilience interventions, which could have contributed to an underestimation of intervention effects. Although its more trait-like character seems to contradict our conceptualization of resilience as an outcome of a dynamic process of adapting to adverse events, we used the BRS as a secondary outcome for two reasons: First, the BRS measures the ability to bounce back or recover from stress and tackles the process of positive adaptation^84^, which is in line with our conceptualization of resilience. Second, the BRS is a widely used outcome in resilience research and respective intervention studies, and results of our studies could therefore be compared to previous and future studies and could also be included in systematic reviews and meta-analyses.

Given that the effect on mental distress was attenuated at follow-up and not observed in ITT analyses, results suggest that improvements were associated with active intervention use and continuous transfer to daily life. Adherence was limited, with participants completing on average 5 modules and only 37% finishing the whole intervention. As this intervention was structured consecutively and resilience factors were targeted in later modules, it is perhaps unsurprising that significant effects were not observed among participants not adhering to the complete intervention as they only completed introductory modules. Further, based on feedback questionnaires, participants reported infrequent use of intervention exercises. Whereas 54% to 77% reported using exercises in daily life in the weekly questionnaires, only 15.38% indicated that they often adopted exercises in the overall post-intervention feedback. Low intervention engagement is a key challenge in online interventions and likely lowers intervention efficacy^38,85,86^. Previously, active engagement and regular practice have been associated with better intervention outcomes for depressive and anxiety symptoms^86,87^. Similarly, a study found associations with changes in mindfulness, mental health and well-being following a mindfulness intervention^88^. It can be expected that regular practice is also associated with the development and improvement of other resilience factors. Hence, low levels of regular practice could have resulted in finding no intervention effect on self-reported resilience and resilience factors. Further, Jayewardene and colleagues^89^ imply that, compared to clinical samples, participants’ motivation and perceived need in non-clinical samples are presumably lower in the absence of significant psychological strain, limiting intervention adherence and engagement.

Compared to in-person settings, high dropout rates and low adherence have been identified as major limitations of online interventions^39^. Several studies on online resilience trainings^90–92^ included human support (e.g. feedback, face-to-face, phone-call, online forum) or offered it upon request to address these challenges^93^. This could have contributed to the variability in observed intervention effects and usage between previous studies and the present results. Guided interventions have been associated with higher levels of adherence and better intervention outcomes than unguided programs^38^. However, heterogeneity in the quantity and type of guidance across studies limits consistent conclusions about the effectiveness of support in online interventions. In our study, the low adherence rate was further exacerbated by the structure of the intervention, as modules were only unlocked upon successful completion of the previous module within a specific time window (1 week). This also meant that no modules could be skipped; in other words, if a participant was unable to take part in a module—for example, due to illness or a lack of time—they could not proceed to the next module.

Contrary to the literature, the number of completed modules did not significantly moderate changes in targeted outcomes, except for optimism. This inconsistency may reflect methodological differences, as prior studies included multiple usage metrics (e.g., number of logins/exercises, time per module)^94^, whereas this study relied solely on the number of completed modules. However, it has been suggested that the predictive value of the mere number of completed modules on intervention benefits is limited as change in outcomes is not solely attributed to the exposure but rather to active intervention engagement and regular practice over time^95^. Therefore, metrics to capture the implementation of exercises may serve as better indicators of long-term improvements following the intervention, however, with the used LMS we were not able to acquire such data.

Despite the discussed opportunities of e-mental health, concerns regarding online interventions should equally be considered in future research and intervention development. These may include concerns regarding data security and privacy, adverse events, social inequalities in accessing those services, and feasibility of e-mental health for specific populations (e.g., severe mental illness)^96–98^. Therefore, we investigated negative effects that might be encountered by participants^68^. In general, participants reported positive intervention effects far more frequently than negative effects. However, reports of negative effects especially highlighted the need to address data protection concerns and secure confidentiality.

Previous studies on online interventions have shown several methodological limitations. This study overcame some of these shortfalls by incorporating the recommendations for (online) resilience intervention studies^44^ including a priori sample size planning, assessment of inclusion and exclusion criteria, baseline assessment of demographic and outcome variables, high psychometric quality of questionnaires, randomization, assessment of user satisfaction, participation flow, usage and qualitative feedback. Nevertheless, important limitations should be acknowledged.

First, this study was not pre-registered. However, all methodological decisions, including sample size, inclusion criteria, and the type of statistical analyses, were determined prior to data collection and analyses. No post hoc changes were made to the analysis plan without explicit annotation in the results section. To ensure replicability and transparency, we provide the code.

Moreover, although the sample was typical for German university students, women were overrepresented and completed significantly more modules than males, potentially limiting the generalizability of results. Although women show a higher prevalence of mental illness than men in student populations^99^ and might therefore have a higher demand for resilience interventions, further research on barriers and help-seeking behaviour of men in student populations is needed^100^. Further, around three quarters of students were from 4 faculties (i.e., Social Sciences, Media, and Sports; Law, Management and Economics; Medicine; Philosophy and Philology) leaving students from natural science, technology, engineering, mathematics underrepresented. These shortcomings in the representative composition of our sample point to a self-selection bias that also affects the generalizability of results as students interested in resilience and mental health are probably also more interested to participate in interventions and therefore might differ from non-participants. Another limitation is the absence of race/ethnicity data. Given that race and ethnicity can be associated with differential access to psychological resources, exposure to stressors, and responsiveness to intervention^101^, this may limit the generalizability of our findings. Future research should include these variables to ensure interventions are equitable and effective across diverse populations. As this sample was a convenience sample from a specific university, future studies implementing the program at different universities could help to increase the external validity and generalizability of the results.

Methodologically, the use of a CG might be associated with risks to internal validity and may result in an overestimation of intervention effects^102^. To counteract this and compare intervention effects to other (established) trainings (e.g., face-to-face, other online interventions), future trials should consider adopting an active control group design or head-to-head trials.

Moreover, all outcomes were self-reported and neither participants nor researchers were blinded. While self-assessment is most suitable for measuring the included constructs, it is crucial to acknowledge that they can be subjectively biased and open to socially desirable reporting or expectation effects. Regarding the measurement time points, baseline and follow-up were carried out during the semester break while the post-test took place during the semester. Different circumstances and demands (e.g., exams) at the respective time points may have had an influence on study results. Finally, drop-out at post-test (26.85%) and follow-up (40.28%) should be mentioned as a limitation even though dropout rates were comparable to other online intervention studies^39,103^. Further, the study experienced a decline of user engagement in the IG over the eight modules. Insights around attrition are limited since reasons for dropout were not directly collected. Reminder emails were insufficient to sustain participant engagement throughout the intervention.

Notwithstanding the limitations, this study constitutes a valuable contribution to the development of online resilience interventions for university students and has several implications for further research, practice and intervention development. First, *Resitra* was not specifically designed for university students. Future intervention development may benefit from tailoring content to student’s needs and predictors of resilience and mental health in this population. Future trials should include specific measures to capture academic stressors. Given that previous studies found associations of resilience with better academic outcomes and functioning^103^, more research is needed to explore possible effects of *Resitra* on academic outcomes.

While excluding students with a diagnosed severe mental disorder (e.g., schizophrenia, post-traumatic stress disorder) or currently receiving treatment ensures internal validity, this might have affected intervention effects as students with higher symptom burden remain unexamined. Considering the low baseline symptom severity of the present sample, future studies examining subgroup-specific intervention preferences and needs, particularly among students with elevated symptom severity, are needed to help identify populations most likely to benefit.

While our results indicated that attitudes toward psychological online interventions might determine dropouts, further predictors of attrition in online interventions remain to be explored. The suggested association of intervention use, and efficacy highlights the need for a detailed understanding of user engagement. Other user metrics that are more predictive in change of behaviour and adoption of skills than number of completed modules (e.g., average time spent per exercise/module) should be examined in future research. Further, possibilities to increase user engagement and adoption of new behaviours should be explored. Previous research provides evidence for enhanced adherence and better outcomes in guided online interventions compared to self-guided programs^26,28,104^. As support might personalize intervention components to specific life circumstances of the participants^38^ this might enable participants to apply targeted skills. Concluding from the feedback that participants simply forgot to adopt exercises into daily life, additional support (e.g., personalized reminders, face-to-face support, virtual synchronous communication platforms)^26^ could also increase adherence and engagement and remind participants of regular practice. Future studies should compare guided versus unguided versions of *Resitra* to examine the impact of support on adherence and intervention effects.

Given the association between satisfaction and module completion, it is important to explore ways to promote satisfaction to ultimately increase adherence and adoption of exercises into daily life. Previously, gamification has been suggested as an opportunity in online interventions to foster adherence, engagement and user motivation^31,105,106^. Recently, we developed a new platform (https://resilir.eu) incorporating gamification into *Resitra*. Exploring the effect of gamification on intervention outcomes, satisfaction, adherence and adoption of exercises in future research and drawing comparisons to the findings in this study which used a non-gamified approach, will provide valuable insights for further intervention development. As satisfaction was associated with better intervention outcomes and greater psychosomatic symptom reduction in previous studies^67,107^, this might also increase intervention effectiveness. Qualitative feedback further suggested that participants took more time than anticipated for each module. Allowing participants to complete exercises at their own pace and prioritize modules according to their needs and interests could optimize intervention use and satisfaction. Involving participants in iterative development and testing, consistent with participatory research approaches, may also improve satisfaction and intervention outcomes^108,109^.

Regarding practical implications, these findings provide evidence for the feasibility of online interventions in reducing mental distress among university students under optimal adherence conditions. Student health services may use such interventions to address increasing demands and waiting times (e.g., 16 to 56 days at Johannes Gutenberg-University from 2016 to 2021)^110^ for counselling by offering self-guided online resilience programs as resource-efficient first line support within a stepped-care approach^2^.

## Conclusion

Online resilience interventions represent a promising and rapidly evolving approach to reduce stress-related mental health problems and overcome challenges related to the accessibility of adequate treatment. This study contributes to the growing research on online resilience interventions, demonstrating the feasibility of *Resitra* in reducing mental distress among university students adhering to the intervention protocol. However, the lack of intervention effects in ITT analyses and on other outcomes as well as methodological constraints and insufficient transfer of learned skills, limit the strength of evidence and remain challenges for future studies. Further research and intervention development is needed to optimize the design and implementation of *Resitra* in terms of user engagement and effectiveness.

## Supplementary Information

Below is the link to the electronic supplementary material.

## Data Availability

The datasets generated during and/or analysed during the current study are available from the corresponding author on reasonable request.

## Footnotes

1 Since one data set was excluded due to invalid data (see Fig. 1), the analyses only took into account 109 data records.
