# Supplementary material for: Efficacy of a self-guided online resilience intervention for improving mental health among university students: A randomized controlled trial
Source: Sci Rep. 2026 Sep 2;16:27557. doi: 10.1038/s41598-026-67088-7 (PMC13538386; doi:10.1038/s41598-026-67088-7)
Supplement: Supplementary file 1 [file 41598_2026_67088_MOESM1_ESM.pdf]

Supplementary material

Supplement S1: Description of intervention modules and its content

Table S1: *Resitra modules and their objectives*

	Module	Objective
1	Introduction	Introduction to the training program and its structure. Reflection on goals and expectations regarding the program.
2	Stress and resilience	Understanding the concept of resilience and stress and how they are related. Reflection on personal experiences (e.g., individual stressors) and introduction on how to increase resilience and decrease stress (e.g., including positive activities into everyday life).
3	Optimism	Understanding realistic optimism and its effects on emotions, behaviour, individual resilience and health. Promoting optimism with various practical exercises (e.g., visualisation).
4	Mindfulness	Understanding the concept of mindfulness, how mindfulness and resilience are related and scientific findings on this topic. Training in mindfulness exercises (e.g., body scan, breathing meditation).
5	Values and sense of coherence	Introduction on values and sense of coherence and their relation to resilience. Identification of personal values and learning how they can be implemented in everyday life.
6	Acceptance	Learning what constitutes an accepting attitude. Combination of stories that users can reflect upon and practical exercises to increase acceptance.
7	Self-compassion	Understanding the concept of self-compassion, how it differs from self-esteem and what the effects of self-compassion are. Learning how to train oneself in self-compassion and how it can be used to strengthen resilience (e.g., self-compassion meditation).
8	Conclusion	Reflection on the participants' progress and what they have learned. Identifying early signals of stress and planning how to implement some of the trained strategies and skills in everyday life.

Supplement S2: Reliabilities of each outcome measure at each measurement point

Table S2: *Reliabilities (Cronbach's α and McDonald's Omega) for scales at pre- and post-test and follow-up*

	Reliabilities					
Scale	Baseline (N = 216)		Post-test (n = 158)		Follow-Up (n = 129)	
	α	ω	α	ω	α	ω
	Primary outcome					
Mental distress (GHQ-12)	0.88	0.88	0.85	0.86	0.87	0.87
	Secondary outcomes					
Resilience (BRS)	0.84	0.84	0.82	0.83	0.83	0.83
Perceived stress – self-efficacy (PSS 2+2)	0.70	0.70	0.72	0.72	0.73	0.73
Perceived stress – helplessness (PSS 2+2)	0.63	0.63	0.65	0.65	0.71	0.71
	Targeted resilience factors					
Optimism (SOP-2)	0.89	0.89	0.87	0.87	0.87	0.87
Mindfulness (MAAS)	0.90	0.90	0.90	0.91	0.91	0.91
Sense of Coherence (SOC-29)	0.90	0.90	0.89	0.90	0.90	0.90
Meaning (CIT)	0.81	0.82	0.81	0.82	0.80	0.81
Acceptance (CERQ)	0.83	0.83	0.88	0.88	0.87	0.87
Self-Compassion (SCS-D)	0.94	0.94	0.94	0.94	0.95	0.95
	Covariates and moderators					
Stressor exposure (MIMIS)	---	---	---	---	---	---
Attitudes towards online interventions (APOI)	0.79	0.80	---	---	---	---
Satisfaction with intervention (CSQ-I)	---	---	0.93[a]	0.93[a]	---	---

Note. [a]n = 79 (only intervention group). No reliability measures for MIMIS available.

Supplement S3: Demographic data of sample for PP analyses

Table S3: *Demographic data of sample of PP analyses*

	All (N = 151)		*IG (n = 70)*		*WLCG (n = 81)*		*t-test/ Chi²-test*
	N/ M [95%-CI]	*%/ Range*	*N/ M [95%-CI]*	*%/ Range*	*N/ M [95%-CI]*	*%/ Range*	*p*
Age	23.89 [23.29; 24.48]	19 - 49	24.40 [23.36; 25.44]	20 - 49	23.44 [22.80; 24.09]	19 - 34	.123
Gender							.373
Female	128	84.77	62	88.57	66	81.48	
Male	22	14.57	8	11.43	14	17.28	
non-binary	1	0.66	0	0.00	1	1.23	
Education							.133
A level	98	64.90	42	60.00	56	69.14	
Bachelor	47	31.13	27	38.57	20	24.69	
Master	3	1.99	1	1.43	2	2.47	
First state exam	3	1.99	0	0.00	3	3.70	
Faculty at JGU							.025
Catholic and Protestant Theology	1	0.66	0	0.00	1	1.23	
Social Sciences, Media, and Sports	47	31.13	22	31.43	25	30.86	
Law, Management and Economics	31	20.53	17	24.29	14	17.28	
Medicine	16	10.60	5	7.14	11	13.58	
Philosophy and Philology	21	13.91	14	20.00	7	8.64	
Translation Studies, Linguistics, Cultural Studies	2	1.32	1	1.43	1	1.23	
History and Cultural Studies	5	3.31	3	4.29	2	2.47	
Physics, Mathematics, and Computer Science	7	4.64	0	0.00	7	8.64	
Chemistry, Pharmaceutical Sciences, Geography and Geosciences	7	4.64	1	1.43	6	7.41	
Biology	7	4.64	3	4.29	4	4.94	
Mainz School of Music	3	1.99	3	4.29	0	0.00	
Mainz Academy of Fine Arts	1	0.66	1	1.43	0	0.00	
Other	3	1.99	0	0.00	3	3.70	

Supplement S4: Correlations at baseline (t0) between primary and secondary outcomes, targeted resilience factors and covariates

Table S4: *Correlations at baseline (t_0) between primary and secondary outcomes, targeted resilience factors and covariates*

Variable	1	2	3	4	5	6	7	8	9	10	11	12
Primary outcome												
1. Mental distress												
Secondary outcomes												
2. Resilience (BRS)	-.50**											
3. Perceived stress – self-efficacy	-.62**	.49**										
4. Perceived stress – helplessness	.54**	-.43**	-.48**									
Targeted resilience factors												
5. Optimism	-.45**	.46**	.52**	-.46**								
6. Mindfulness	-.47**	.40**	.43**	-.39**	.33**							
7. Sense of Coherence	-.58**	.61**	.67**	-.49**	.62**	.57**						
8. Meaning	-.40**	.29**	.53**	-.18**	.38**	.33**	.60**					
9. Acceptance	-.22**	.28**	.27**	-.11	.23**	.04	.18**	.19**				
10. Self-compassion	-.62**	.63**	.59**	-.51**	.54**	.48**	.75**	.42**	.29**			
Covariates and moderators												
11. Stressor exposure	.32**	-.24**	-.16*	.33**	-.11	-.39**	-.29**	-.03	.01	-.25**		
12. Attitudes towards online interventions	-.06	.07	.07	-.06	.04	.07	.13	.10	.09	.11	-.08	
13. Satisfaction with intervention	-.09	.12	.21	-.09	.15	.26*	.22*	.32**	.02	.28**	.03	.30**

Note. * indicates $p < .05$. ** indicates $p < .01$.

Supplement S5: Descriptive data of Client Satisfaction Questionnaire (CSQ-I)

Table S5: *Descriptive statistics for satisfaction with intervention (CSQ-I) within the IG at t1*

Item	*M*	*SD*
The training I attended was of high quality	3.49	0.61
I received the kind of training I wanted	3.24	0.66
The training has met my needs	3.04	0.72
I would recommend this training to a friend, if he or she were in need of similar help.	3.32	0.82
I am satisfied with the amount of help I received through the training.	3.28	0.75
The training helped me deal with my problems more effectively.	3.03	0.76
In an overall, general sense, I am satisfied with the training.	3.43	0.71
I would come back to such a training if I were to seek help again.	3.19	0.83
Overall	3.25	0.60

Note. *N* = 79; *M* = mean; *SD* = standard deviation; range = 1-4.

Supplement S6: Results of intention-to-treat analyses

Table S6: Results of intention-to-treat analyses of primary and secondary outcomes: Overall group x time effects and specific group x time effects for post-intervention and follow-up (3 months after completion of intervention)

**Outcome**		**b**	**SE**	**95% CI**	**df**	**t**	***p***	
				Primary outcome				
Mental Distress (GHQ)	Overall	-0.72	0.68	[-2.07; 0.64]	236.50	-1.04	.298	-
	9	-1.09	0.92	[-2.90; 0.72]	130.12	-1.20	.234	-
	21	-0.54	0.97	[-2.47; 1.38]	92.80	-0.56	.577	-
				Secondary outcomes				
Resilience (BRS)	Overall	0.03	0.06	[-0.09; 0.14]	366.24	0.49	.626	.976
	9	0.02	0.07	[-0.12; 0.16]	349.13	0.31	.759	-
	21	0.06	0.08	[-0.10; 0.22]	111.98	0.74	.460	-
Perceived stress – self-efficacy	Overall	0.09	0.08	[-0.06; 0.25]	187.13	1.23	.219	.976
	9	0.08	0.10	[-0.12; 0.27]	125.28	0.78	.436	-
	21	0.09	0.09	[-0.09; 0.26]	259.07	0.97	.334	-
Perceived stress - helplessness	Overall	-0.05	0.09	[-0.22; 0.13]	194.38	-0.54	.587	.976
	9	-0.01	0.10	[-0.21; 0.20]	282.66	0.32	.748	-
	21	-0.07	0.11	[-0.28; 0.15]	180.07	-0.07	.948	-
				Targeted resilience factors				
Optimism	Overall	0.07	0.10	[-0.13; 0.26]	254.13	-0.60	.550	.976
	9	0.06	0.12	[-0.17; 0.29]	256.10	0.51	.608	-
	21	0.03	0.13	[-0.22; 0.28]	135.24	0.22	.827	-
Mindfulness	Overall	0.01	0.08	[-0.14; 0.15]	228.82	0.09	.932	.976
	9	0.03	0.09	[-0.15; 0.20]	252.75	0.28	.778	-
	21	-0.00	0.10	[-0.19; 0.19]	120.18	-0.01	.990	-
Sense of coherence	Overall	0.04	0.05	[-0.06; 0.13]	346.91	0.79	.429	.976
	9	0.04	0.06	[-0.07; 0.16]	206.45	0.74	.458	-
	21	0.07	0.06	[-0.04; 0.19]	183.78	1.23	.219	-
Meaning	Overall	-0.00	0.07	[-0.14; 0.14]	163.37	-0.03	.976	.976
	9	-0.00	0.08	[-0.18; 0.15]	274.41	-0.04	.970	-
	21	-0.00	0.09	[-0.20; 0.17]	95.24	-0.01	.989	-
Acceptance	Overall	0.18	0.10	[-0.01; 0.37]	229.51	1.82	.069	.476
	9	0.13	0.13	[-0.12; 0.38]	433.69	1.04	.298	-
	21	0.25	0.13	[0.00; 0.50]	129.51	1.99	**.048**	-
Self-compassion	Overall	0.09	0.05	[-0.00; 0.18]	319.25	1.92	.056	.711
	9	0.10	0.06	[-0.02; 0.22]	173.21	1.68	.095	-
	21	0.08	0.06	[-0.05; 0.20]	135.04	1.24	.219	-

Note. Week 9 = post-intervention; week 21 = follow-up (3 months after completion of the intervetion). p_{raw} = unadjusted p-values, p_{hommel} = Hommel-adjusted p-values (as sensitivity analysis for secondary outcomes Unadjusted p-values < .05 are shown in bold.

Supplement S7: Descriptive data of Inventory of Negative Effects of Online-Interventions (INEP-ON)

Table S7: *Descriptive statistics for Inventory of Negative Effects of Online-Interventions (INEP-ON) within the IG at t1*

		Overall			Intervention				Life circumstances			
	Item	*M*	*SD*	n	N	%	*M*	*SD*	N	%	*M*	*SD*
1	Since completing the online intervention, I feel worse/ better.	0.96	1.16	68	28	41.18	1.46	0.79	40	58.82	0.65	1.31
2	Since completing the online intervention, I find it harder/easier to trust others compared to before.	0.24	0.88	52	15	28.85	0.47	0.64	37	71.15	0.16	0.58
3	Among the events from my past, since completing the online intervention, compared to before, I suffer more/ less.	0.54	0.94	58	29	50.00	0.97	0.99	29	50.00	0.24	0.99
4	Since completing the online intervention, I have experienced in my relationship, compared to before more/ fewer conflicts.	0.24	0.87	54	15	27.78	0.20	1.08	39	72.22	0.28	0.94
5	My relationship with my family has worsened/ improved since completing the online intervention compared to before.	0.33	0.98	54	11	20.37	0.91	1.30	43	79.63	0.35	1.04
6	My relationship with my friends has worsened/ improved since completing the online intervention.	0.25	0.99	55	18	32.73	0.89	0.96	37	67.27	0.03	1.07
7	Feelings of loneliness have increased/ decreased during the use of the online intervention.	0.33	1.31	62	23	37.10	0.87	1.10	39	62.90	0.08	1.48
8	My hobbies and social contacts have decreased/ increased during the use of the online intervention.	0.42	1.06	56	19	33.93	0.63	0.96	37	66.07	0.49	1.26
9	The ways of thinking that I learned in the online intervention were harmful/ helpful.	1.91	0.91	68	64	94.12	2.03	0.82	4	5.88	0.50	1.00
10	My motivation to start psychotherapy was decreased/ increased.	0.38	0.84	61	37	60.66	0.65	0.95	24	39.34	0.17	0.48
11	I found the guide/ moderator in the online intervention to be disturbing/ supportive.	1.46	1.38	60	56	93.33	1.63	1.32	4	6.67	0.50	1.73
12	I am afraid that my classmates/fellow students/colleagues might find out that I have used an online intervention.	0.03	0.16	52	21	40.38	0.10	0.30	31	59.62	0.00	0.00
13	I have had problems with insurance companies (e.g., life insurance) or am afraid that problems might arise.	0.08	0.31	50	15	30.00	0.20	0.41	35	70.00	0.09	0.37

14	I am more worried about money than before.	0.52	0.80	56	11	19.64	0.09	0.30	45	80.36	0.80	0.84
15	I feel dependent on the online intervention.	0.08	0.27	48	21	43.75	0.19	0.40	27	56.25	0.04	0.19
16	I find it more difficult to make important decisions on my own.	0.39	0.76	50	13	26.00	0.54	0.97	37	74.00	0.51	0.77
17	I have had relationship problems with my partner.	0.43	0.81	53	11	20.75	0.64	0.81	42	79.25	0.55	0.97
18	I have had prolonged periods of feeling bad.	0.87	0.95	58	10	17.24	0.60	1.07	48	82.76	1.23	0.90
19	I have changed for the worse as a person.	0.14	0.38	50	17	34.00	0.12	0.33	33	66.00	0.24	0.50
20	I have had suicidal thoughts/intentions for the first time.	0.03	0.16	47	10	21.28	0.20	0.42	37	78.72	0.00	0.00
21	I have consumed more alcohol/drugs than before.	0.15	0.46	47	8	17.02	0.00	0.00	39	82.98	0.26	0.55

Note. *N* = 79, *M* = mean; *SD* = standard deviation; Range Items 1 – 11 = -3; +3 with higher values indicating better outcomes; Range items 12 – 21: 0; 3 with lower values indicating better outcomes.

Figure S7.1: *INEP-ON positive and negative effects attributed to the intervention or life circumstance (numbers in bars refer to participants per category)*

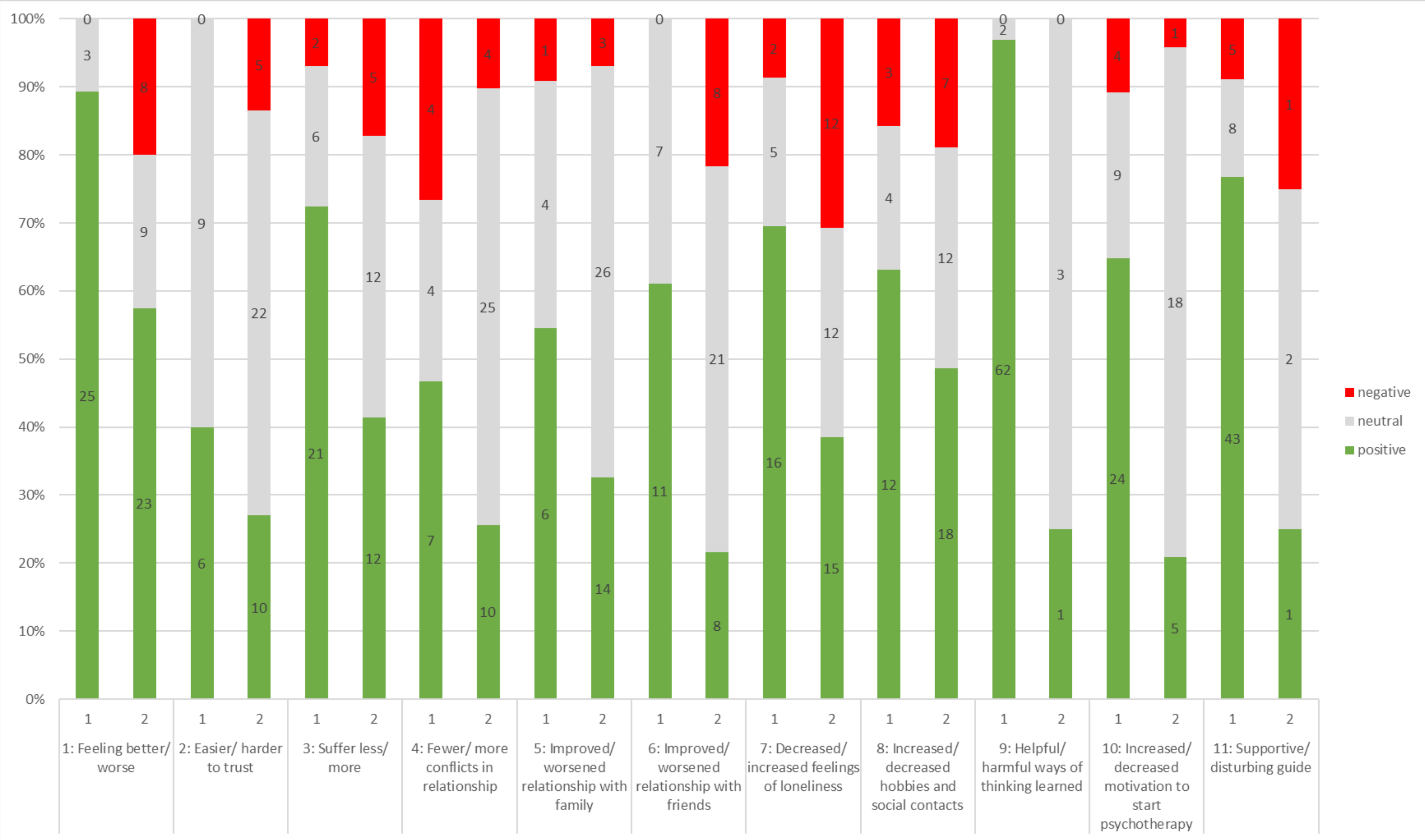

Note. 1 = Effects attributed to the intervention; 2 = Effects attributed to life circumstances.

Figure S7.2: *INEP-ON negative effects attributed to the intervention or life circumstance (numbers in bars refer to participants per category)*

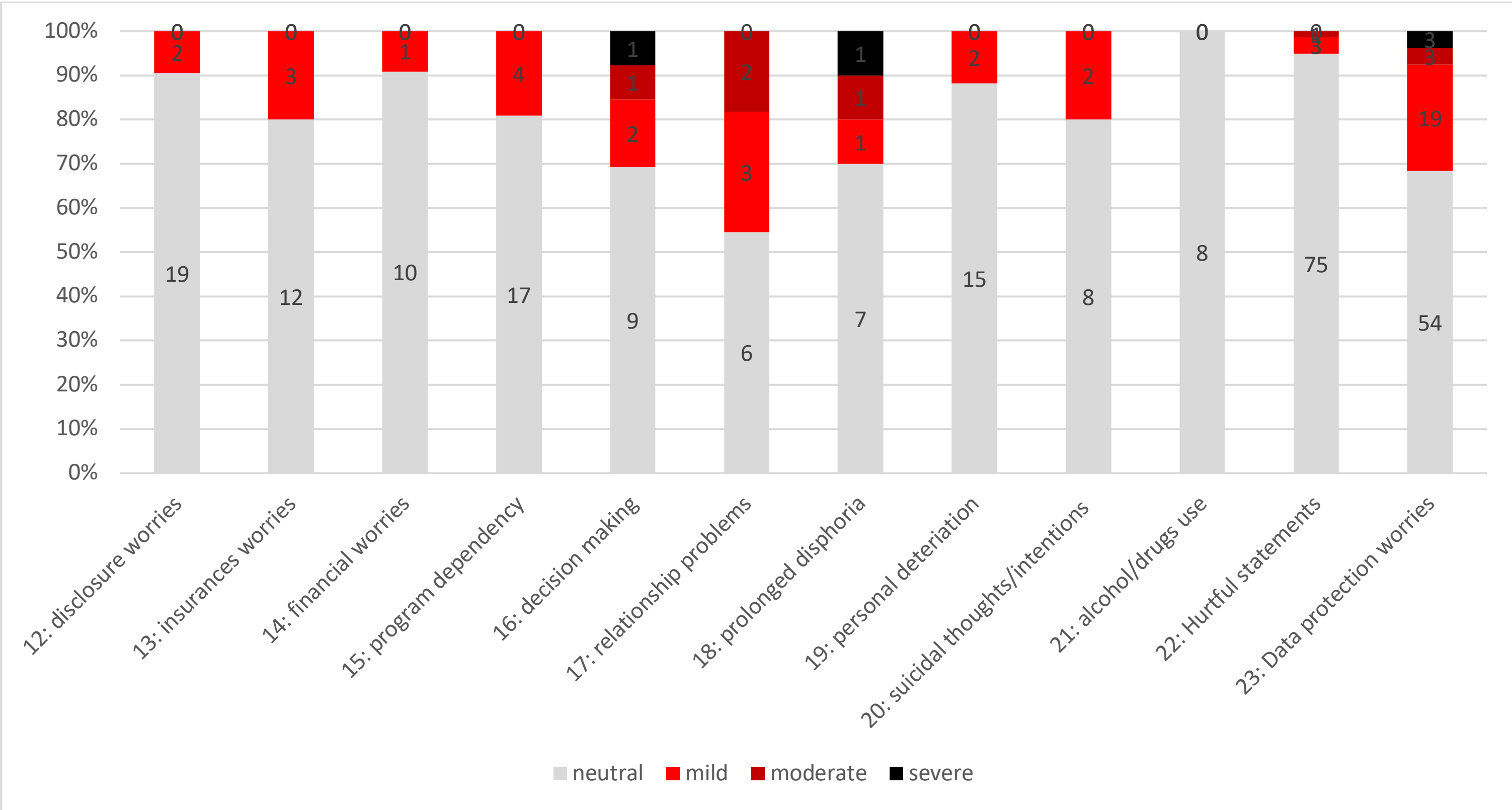

Supplement S8: TIDieR and CONSORT checklist

Table S8.1: TIDieR checklist

TIDieR
Template for Intervention Description and Replication

The TIDieR (Template for Intervention Description and Replication) Checklist*:

Information to include when describing an intervention and the location of the information

Item number	Item	Where located	
		Primary paper (page or appendix number)	Other (details)
	**BRIEF NAME**		
**1.**	Provide the name or a phrase that describes the intervention.	1; 15	
	**WHY**		
**2.**	Describe any rationale, theory, or goal of the elements essential to the intervention.	5; 6	
	**WHAT**		
**3.**	Materials: Describe any physical or informational materials used in the intervention, including those provided to participants or used in intervention delivery or in training of intervention providers. Provide information on where the materials can be accessed (e.g. online appendix, URL).	Supplement S1	
**4.**	Procedures: Describe each of the procedures, activities, and/or processes used in the intervention, including any enabling or support activities.	6 – 8; Supplement S1	

	**WHO PROVIDED**		
**5.**	For each category of intervention provider (e.g. psychologist, nursing assistant), describe their expertise, background and any specific training given.	N/A self-guided digital intervention	______________
	**HOW**		
**6.**	Describe the modes of delivery (e.g. face-to-face or by some other mechanism, such as internet or telephone) of the intervention and whether it was provided individually or in a group.	7 - 8	______________
	**WHERE**		
**7.**	Describe the type(s) of location(s) where the intervention occurred, including any necessary infrastructure or relevant features.	N/A digital intervention	______________
	**WHEN and HOW MUCH**		
**8.**	Describe the number of times the intervention was delivered and over what period of time including the number of sessions, their schedule, and their duration, intensity or dose.	8 – 9; 15	______________
	**TAILORING**		
**9.**	If the intervention was planned to be personalised, titrated or adapted, then describe what, why, when, and how.	N/A	______________
	**MODIFICATIONS**		
**10.**	If the intervention was modified during the course of the study, describe the changes (what, why, when, and how).	N/A	______________
	**HOW WELL**		
**11.**	Planned: If intervention adherence or fidelity was assessed, describe how and by whom, and if any strategies were used to maintain or improve fidelity, describe them.	23	______________
**12.**	Actual: If intervention adherence or fidelity was assessed, describe the extent to which the intervention was delivered as planned.	23	______________

Table S8.2: CONSORT 2025 checklist of information to include when reporting a randomised trial*

Section / Topic	No	CONSORT 2025 checklist item description	Reported on page no.
**Title and abstract**			
Title and structured abstract	1a	Identification as a randomised trial	1
	1b	Structured summary of the trial design, methods, results, and conclusions	3
**Open science**			
Trial registration	2	Name of trial registry, identifying number (with URL) and date of registration	8
Protocol and statistical analysis plan	3	Where the trial protocol and statistical analysis plan can be accessed	Study protocol
Data sharing	4	Where and how the individual de-identified participant data (including data dictionary), statistical code and any other materials can be accessed	19
Funding and conflicts of interest	5a	Sources of funding and other support (e.g., supply of drugs), and role of funders in the design, conduct, analysis and reporting of the trial	44
	5b	Financial and other conflicts of interest of the manuscript authors	44
**Introduction**			
Background and rationale	6	Scientific background and rationale	4 - 6
Objectives	7	Specific objectives related to benefits and harms	6
**Methods**			
Patient and public involvement	8	Details of patient or public involvement in the design, conduct and reporting of the trial	N/A
Trial design	9	Description of trial design including type of trial (e.g., parallel group, crossover), allocation ratio, and framework (e.g., superiority, equivalence, non-inferiority, exploratory)	6 - 8
Changes to trial protocol	10	Important changes to the trial after it commenced including any outcomes or analyses that were not prespecified, with reason	N/A
Trial setting	11	Settings (e.g., community, hospital) and locations (e.g., countries, sites) where the trial was conducted	7
Eligibility criteria	12a	Eligibility criteria for participants	8 - 9

	12b	If applicable, eligibility criteria for sites and for individuals delivering the interventions (e.g., surgeons, physiotherapists)	N/A
Intervention and comparator	13	Intervention and comparator with sufficient details to allow replication. If relevant, where additional materials describing the intervention and comparator (e.g., intervention manual) can be accessed	36; Supplement S1
Outcomes	14	Pre-specified primary and secondary outcomes, including the specific measurement variable (e.g., systolic blood pressure), analysis metric (e.g., change from baseline, final value, time to event), method of aggregation (e.g., median, proportion), and time point for each outcome	12 - 16
Harms	15	How harms were defined and assessed (e.g., systematically, non-systematically)	16
Sample size	16a	How sample size was determined, including all assumptions supporting the sample size calculation	8
	16b	Explanation of any interim analyses and stopping guidelines	N/A
Randomisation:			
Sequence generation	17a	Who generated the random allocation sequence and the method used	7
	17b	Type of randomisation and details of any restriction (e.g., stratification, blocking and block size)	7
Allocation concealment mechanism	18	Mechanism used to implement the random allocation sequence (e.g., central computer/telephone; sequentially numbered, opaque, sealed containers), describing any steps to conceal the sequence until interventions were assigned	7
Implementation	19	Whether the personnel who enrolled and those who assigned participants to the interventions had access to the random allocation sequence	7
Blinding	20a	Who was blinded after assignment to interventions (e.g., participants, care providers, outcome assessors, data analysts)	7
	20b	If blinded, how blinding was achieved and description of the similarity of interventions	N/A
Statistical methods	21a	Statistical methods used to compare groups for primary and secondary outcomes, including harms	16 - 19
	21b	Definition of who is included in each analysis (e.g., all randomised participants), and in which group	16 - 19
	21c	How missing data were handled in the analysis	16 - 19
	21d	Methods for any additional analyses (e.g., subgroup and sensitivity analyses), distinguishing prespecified from post-hoc	16 - 19
**Results**			
Participant flow, including flow diagram	22a	For each group, the numbers of participants who were randomly assigned, received intended intervention, and were analysed for the primary outcome	11
	22b	For each group, losses and exclusions after randomisation, together with reasons	11
Recruitment	23a	Dates defining the periods of recruitment and follow-up for outcomes of benefits and harms	7 – 8

	23b	If relevant, why the trial ended or was stopped	N/A
Intervention and comparator delivery	24a	Intervention and comparator as they were actually administered (e.g., where appropriate, who delivered the intervention/comparator, how participants adhered, whether they were delivered as intended [fidelity])	20; 23
	24b	Concomitant care received during the trial for each group	N/A
Baseline data	25	A table showing baseline demographic and clinical characteristics for each group	10; 19 - 20
Numbers analysed, outcomes and estimation	26	For each primary and secondary outcome, by group:<br>● the number of participants included in the analysis<br>● the number of participants with available data at the outcome time point<br>● result for each group, and the estimated effect size and its precision (such as 95% confidence interval)<br>● for binary outcomes, presentation of both absolute and relative effect size	
Harms	27	All harms or unintended events in each group	21 – 22, Supplement
Ancillary analyses	28	Any other analyses performed, including subgroup and sensitivity analyses, distinguishing pre-specified from post-hoc	18 - 21
**Discussion**			
Interpretation	29	Interpretation consistent with results, balancing benefits and harms, and considering other relevant evidence	27 - 29
Limitations	30	Trial limitations, addressing sources of potential bias, imprecision, generalisability, and, if relevant, multiplicity of analyses	29 - 32

*We strongly recommend reading this statement in conjunction with the CONSORT 2025 Explanation and Elaboration and/or the CONSORT 2025 Expanded Checklist for important clarifications on all the items. We also recommend reading relevant CONSORT extensions. See www.consort-spirit.org.

Citation: Hopewell S, Chan AW, Collins GS, Hróbjartsson A, Moher D, Schulz KF, et al. CONSORT 2025 Statement: updated guideline for reporting randomised trials. BMJ. 2025; 388:e081123. https://dx.doi.org/10.1136/bmj-2024-081123.

© 2025 Hopewell et al. This is an Open Access article distributed under the terms of the Creative Commons Attribution License (https://creativecommons.org/licenses/by/4.0/), which permits unrestricted use, distribution, and reproduction in any medium, provided the original work is properly cited.
